# Remodeling the bladder tumor immune microenvironment by mycobacterial species with changes in their cell envelope composition

**DOI:** 10.3389/fimmu.2022.993401

**Published:** 2022-10-11

**Authors:** Jordi Senserrich, Sandra Guallar-Garrido, Elisabet Gomez-Mora, Victor Urrea, Bonaventura Clotet, Esther Julián, Cecilia Cabrera

**Affiliations:** ^1^ AIDS Research Institute IrsiCaixa, Institut de Recerca en Ciències de la Salut Germans Trias i Pujol (IGTP), Hospital Germans Trias i Pujol, Universitat Autònoma de Barcelona, Barcelona, Spain; ^2^ Departament de Genètica i de Microbiologia, Facultat de Biociències, Universitat Autònoma de Barcelona, Barcelona, Spain; ^3^ Fundació lluita contra la SIDA, Infectious Diseases Department, Hospital Germans Trias i Pujol, Catalonia, Spain; ^4^ University of Vic-Central University of Catalonia (UVic - UCC), Vic, Spain; ^5^ Consorcio Centro de Investigación Biomédica en Red de Enfermedades Infecciosas (CIBERINFEC), Instituto de Salud Carlos III, Madrid, Spain

**Keywords:** non-muscle invasive bladder cancer, bcg, *Mycobacterium brumae*, immunotherapy, intravesical mycobacterial treatment, tumor immune microenvironment

## Abstract

Intravesical BCG instillation after bladder tumor resection is the standard treatment for non-muscle invasive bladder cancer; however, it is not always effective and frequently has undesirable side effects. Therefore, new strategies that improve the clinical management of patients are urgently needed. This study aimed to comprehensively evaluate the bladder tumor immune microenvironment profile after intravesical treatment with a panel of mycobacteria with variation in their cell envelope composition and its impact on survival using an orthotopic murine model to identify more effective and safer therapeutic strategies. tumor-bearing mice were intravesically treated with a panel of BCG and *M. brumae* cultured under different conditions. Untreated tumor-bearing mice and healthy mice were also included as controls. After mycobacterial treatments, the infiltrating immune cell populations in the bladder were analysed by flow cytometry. We provide evidence that mycobacterial treatment triggered a strong immune infiltration into the bladder, with BCG inducing higher global absolute infiltration than *M. brumae*. The induced global immune microenvironment was strikingly different between the two mycobacterial species, affecting both innate and adaptive immunity. Compared with *M. brumae*, BCG treated mice exhibited a more robust infiltration of CD4^+^ and CD8^+^ T-cells skewed toward an effector memory phenotype, with higher frequencies of NKT cells, neutrophils/gMDSCs and monocytes, especially the inflammatory subset, and higher CD4^+^ T_EM_/CD4^+^ T_reg_ and CD8^+^ T_EM_/CD4^+^ T_reg_ ratios. Conversely, *M. brumae* treatment triggered higher proportions of total activated immune cells and activated CD4^+^ and CD8^+^ T_EM_ cells and lower ratios of CD4^+^ T_EM_ cells/CD4^+^ T_regs_, CD8^+^ T_EM_ cells/CD4^+^ T_regs_ and inflammatory/reparative monocytes. Notably, the mycobacterial cell envelope composition in *M. brumae* had a strong impact on the immune microenvironment, shaping the B and myeloid cell compartment and T-cell maturation profile and thus improving survival. Overall, we demonstrate that the bladder immune microenvironment induced by mycobacterial treatment is species specific and shaped by mycobacterial cell envelope composition. Therefore, the global bladder immune microenvironment can be remodelled, improving the quality of infiltrating immune cells, the balance between inflammatory and regulatory/suppressive responses and increasing survival.

## Graphical Abstract

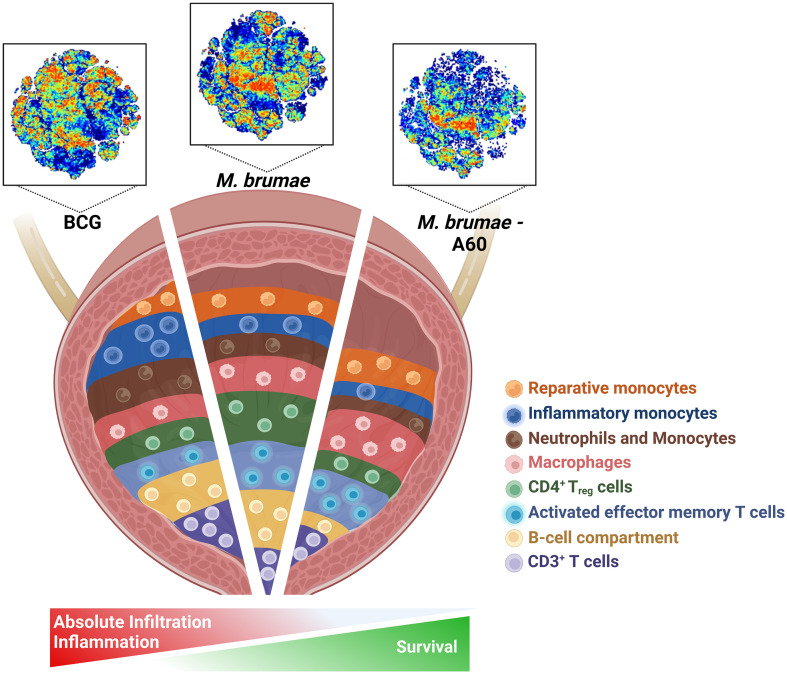

## Introduction

Bladder cancer (BC) is the fourth most common cancer in men, and approximately 75% of BC cases are classified as non-muscle invasive bladder cancer (NMIBC) at initial diagnosis (1). The current gold standard for treatment of high-risk NMIBC is transurethral resection of bladder tumor followed by intravesical bacillus Calmette–Guérin (BCG) instillations as an immunotherapeutic strategy to prevent recurrence and reduce the risk of progression (2). Unfortunately, despite treatment, 30-50% of patients receiving BCG fail to respond, and 10-15% experience progression to muscle-invasive bladder cancer (3). To solve this difficult-to-treat disease many clinical trials are testing the efficacy of innovative immunological and target agents in both BCG unresponsive and naïve patients, such as checkpoint inhibitors, oncolytic viruses, cancer vaccines, tyrosine kinase receptor inhibitors, or other immunostimulatory agents as the IL-15 superagonist N-803 (4, 5).

Intravesical BCG treatment is generally well tolerated, although both local and systemic side effects have been reported (6). To maximize the antitumor effect of BCG and reduce toxicity, several other alternatives, such as the use of genetically modified BCG strains or the combination of BCG with other immunotherapeutic agents, have been widely explored (4, 5, 7), although none have been approved for clinical use. One promising alternative is the use of mycobacteria other than BCG, such as *Mycobacterium brumae* (*M. brumae*), a safe nontuberculous mycobacterium (8) that has been shown to have a potential role in NMIBC treatment in *in vitro* and *in vivo* preclinical studies since it inhibits tumor proliferation and triggers an effective antitumor immune response (9–11).

It is well recognized that the tumor immune microenvironment not only is a key regulator of cancer progression but also plays a crucial role in cancer treatment response; therefore, strategies to optimize the tumor immune microenvironment are being investigated (12). In BC, BCG treatment has been associated with nonspecific antitumor immune response in the bladder mucosa, but despite its long-term use, their exact mechanism of action is not completely understood (13). Several studies have shown that the pretreatment tumor immune microenvironment plays an important role in determining the BCG response in patients (14–18). Reports have also shown that post-BCG tissues are infiltrated with increased numbers of different subpopulations of CD4^+^ and CD8^+^ T-cells and, to a minor extent, other immune populations (17, 18). However, our understanding of the BCG-induced immune landscape remains incomplete, mainly due to the difficulty associated with obtaining tissue samples from patients during treatment. Using animal models, it has been shown that CD4^+^ and CD8^+^ T-cells play a critical role in BCG- and *M. brumae*-induced antitumor activity (10, 19–21), although comprehensive immune profiling has not been reported.

Given the very few advances in therapeutic strategies for early-stage disease over the past two decades, there is a major unmet need for improved intravesical therapies for NMIBC. We very recently showed that the mycobacterial cell-surface is modified by culture conditions, which impacts antitumor immune activity (21, 22). This is relevant since commercially available BCG sub-strains are currently cultured on different medium compositions, which is one of the plausible reasons for the different treatment outcomes observed in NMIBC patients after BCG treatment. The aim of this study was to provide comprehensive immune profiling of the bladder in an orthotopic murine BC model upon mycobacterial treatment using a panel of mycobacteria that displayed changes in their cell envelope due to the culture conditions. Overall, we demonstrated that the global bladder tumor immune microenvironment could be remodelled by improving the balance between inflammatory and regulatory/suppressive responses after mycobacterial treatment. This knowledge may provide clues for the mechanism of action of mycobacteria as a cancer immunotherapy, how to fine-tune the tumor microenvironment to fight cancer more efficiently, and the identification of more effective and less toxic therapeutic strategies.

## Materials and methods

### Bacterial Strains and BC Cell Line

*M. brumae* (American Type Culture Collection, ATCC 51384), and *M. bovis* BCG Connaught strain (ATCC 35745) were grown in Middlebrook 7H10 agar (Difco Laboratories, UK) supplemented with 10% oleic-albumin-dextrose-catalase enrichment medium for 1-4 weeks, respectively. Mycobacteria grown in solid media were used to inoculate three compositions of Sauton culture medium differing on the amino acid source and glycerol concentration: L-asparagine plus 6% glycerol (A60), L-glutamate plus 1.5% glycerol (G15) and L-glutamate plus 6% glycerol (G60) (22).

The murine BC cell line MB49 was maintained in Dulbecco’s modified Eagle’s medium (Gibco, BRL), with 10% fetal bovine serum (FBS) (Lonza, Switzerland), 100 U/mL penicillin G (Laboratorios, Spain) and 100 μg/ml streptomycin (Laboratorio Reig Jofré, Spain).

### Orthotopic model of BC and intravesical treatment

The mouse orthotopic model of BC was generated as described previously (11). Briefly, 8 randomized C57BL/6 female mice (6 to 8-week-old, Charles River Laboratories, Spain) per group were used. Tumors were induced by initial intravesical administration of poly-L-lysine (Sigma-Aldrich) followed by MB49 cells instillation. 24 hours after tumor implantation, mice were intravesically treated with BCG or *M. brumae* grown in different conditions (21). Mycobacterial treatment was performed weekly for four weeks. Mice not inoculated with tumor cells nor with mycobacteria were used as controls. Tumor implantation was ensured due to the presence of blood in urine between days 7 to 10 after induction. Animal behavior and well-being was evaluated daily to avoid unnecessary suffering, and animals were euthanized when it was required. Animals were sacrificed at day 29 ± 1, and bladders were removed in aseptic conditions for immunological characterization. For survival analyses, another set of experiments were performed following the same scheduled as before, but analyzing clinical symptoms until day 60 after tumor induction when surviving animals were sacrificed (21).

### Bladder tissue processing and staining

The immune infiltrate present into the bladder was analyzed by flow cytometry as previously described (10). Briefly, bladders were minced using a scalpel followed by digestion with 0.5 mg/mL collagenase II (Sigma, Spain) in RPMI-5% FBS 1U/mL DNAse I medium at 37°C for two-three successive 30 min-cycles, with continuous shaking. The cell suspension obtained was filtered through a 40-μm disposable plastic strainer (Becton & Dickinson) and pelleted for staining. Cells were labeled using two antibody panels: Panel 1: CD45, CD3, CD4, CD8, CD62L, NK1.1, CD127, CD44, CD25 and TCRγδ; Panel 2: CD45, CD3, CD45R/B220, CD11b, CD11c, Ly6G, Ly6C and F4/80. LIVE/DEAD Fixable Aqua Dead Cell Stain Kit (Invitrogen) was used to determine cell viability. The antibodies used are shown in **Supplementary Table 1**.

### Flow cytometry analysis

After gating on single-cell population, dead cells were excluded and immune cell were selected by morphological parameters and expression of CD45. Absolute cell numbers were obtained by using Perfect-Count Microspheres (Cytognos). Samples were acquired in a Fortessa flow cytometer (Becton & Dickinson), and data were analyzed using FlowJo software (v10.7.1; TreeStar). Boolean gating was used to generate different population subsets analyzed. Analysis and presentation were performed using SPICE version 6.0 (https://niaid.github.io/spice). Global immune profile was done using OMIQ data analysis software (www.omiq.ai). Cells were gated on CD45^+^ singlets and individual files were concatenated and clustered as a whole in each group of animals using the optimized t-distributed stochastic neighbor embedding (opt-SNE) algorithm for dimensional reduction and visualization. The opt-SNE plot of each panel was subdivided into 20 spatially distinct subpopulations using a manual gating strategy. Median fluorescence intensities of each marker across all populations were plotted on a hierarchically cluster heat map.

### Statistical analysis

GraphPad Prism 8.0 software (San Diego, USA) was used for statistical analyses. Immune infiltration data were analyzed using Mann-Whitney tests. Statistical significance was assumed at p values below 0.05.

## Results

### Absolute immune infiltration is species dependent and modulated by cell envelope composition but is not a predictor of survival

**Figure 1:**
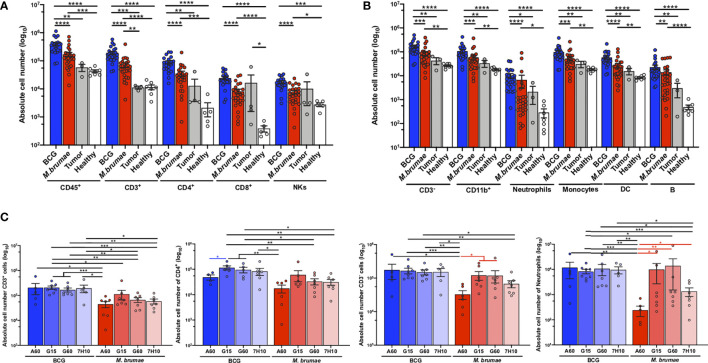
Robust infiltration of immune cells into the bladder after mycobacteria treatment. Absolute counts of **(A)** total live CD45^+^ cells, CD3^+^, CD4^+^ and CD8^+^ T-cells, and NK^+^ cells (CD3^-^NK1.1^+^); and **(B)** CD3^-^ cells, CD11b^+^ myeloid cells (CD3^-^B220^-^CD11b^+^), neutrophils (CD3^-^B220^-^Ly6C^+^Ly6G^+^), monocytes (CD3^-^B220^-^Ly6G^-^CD11b^+^), dendritic cells (CD3^-^B220^-^Ly6G^-^CD11c^+^) and B-cells (CD3^-^B220^+^) were quantified by flow cytometry after bladder digestion of healthy mice (white bars and dots), untreated tumor-bearing mice (grey bars and dots) and mycobacteria-treated mice, either BCG (blue bars and dots) or *M. brumae* (red bars and dots) regardless the medium used to growth mycobacteria. **(C)** Absolute infiltration of total CD3^+^ T-cells, CD4^+^ T-cells, total CD3^-^ cells and neutrophils of mice which received BCG (blue bars) or *M. brumae* (red bars) grown in different culture media. Each dot represents an individual mouse. Data represent the mean (bars) and ± SEM (error bars) and each dot represent an individual mouse. Statistically significant differences between the different BCG and *M. brumae* mycobacteria (intrastrain) are shown as blue and red lines, respectively. Interstrain differences or with the untreated groups (healthy and tumor) are shown in black. Differences were tested using Mann-Whitney U nonparametric test. *p ≤ 0.05; **p ≤ 0.01; ***p ≤ 0.001; ****p ≤ 0.0001.

A murine orthotopic model of BC was used to deeply characterize the immune infiltration in bladder tumors after intravesical treatment with a panel of mycobacteria that displayed changes in their cell envelope lipidomic profile induced by growth in different conditions (21). In comparison with no treatment, BCG and *M. brumae* treatments resulted in a robust and significant increase in the absolute number of immune cells infiltrating the bladder, including both T-cells and non-T-cells, with significantly higher infiltration in BCG-treated animals than in *M. brumae*-treated animals (**Figures 1A, B**; gating strategy **Supplementary Figures 1**, **2**). No correlation was found between total immune infiltration and survival, with longer survival in *M. brumae*- than in BCG-treated animals (21) (**Supplementary Figure 3**).

There were no statistically significant differences in the total infiltrating cells in different immune populations between the untreated groups (healthy and tumor), except for higher CD8^+^ T-cell infiltration observed after tumor induction (**Figure 1A**). Several other populations were increased in the tumor group, but no significant differences were reached, most likely because of the low number of animals in the untreated tumor group that survived to the end of the experiment (**Figures 1A, B**).

Between BCG and *M. brumae* grown in different culture media, we observed that treatment with *M. brumae*-A60 (*M. brumae* grown in A60 medium) induced significantly lower infiltration of most immune populations than all BCG and the rest of the *M. brumae* treatments (**Figure 1C** and **Supplementary Figure 4**). We have previously shown that *M. brumae*-A60 treated mice have a prolonged survival than the rest of *M. brumae* and BCG treated mice, although no significant differences were reached (Reference 21 and **Supplementary Figure 3**). This data further confirms the lack of association between total immune infiltration and survival. When BCG-induced immune infiltration was analysed, there were significant decreases in the numbers of infiltrating CD4^+^ T-cells and B-cells in the BCG-A60 group in comparison with the group BCG-G60 and BCG-G15 groups, respectively (**Figure 1C** and **Supplementary Figure 3**).

### Mycobacterial cell envelope composition lead to global changes in the immune microenvironment in bladder tumors

**Figure 2:**
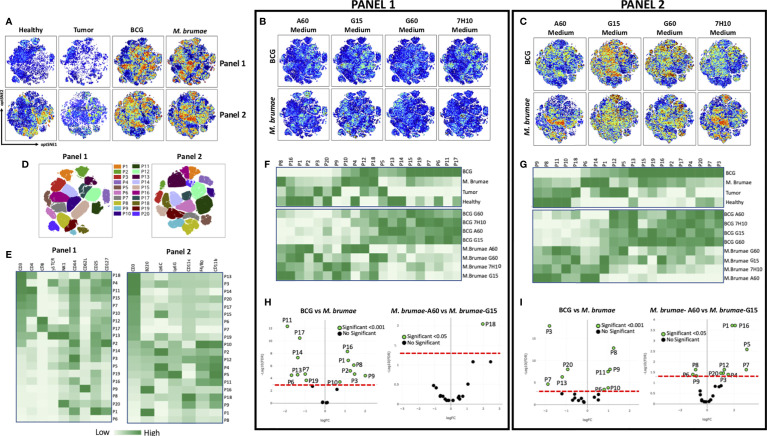
Global profile of bladder infiltrating immune cells. opt-SNE was used for the analysis of the immune infiltration into the bladder of healthy, non-treated and mycobacteria-treated tumor-bearing mice. Cells were stained using two panels of antibodies (Supplementary Table 1), measured by flow cytometry and analyzed using OMIQ software. **(A)** opt-SNE maps showing expression intensity of all markers from both antibody panels of concatenated files for all healthy animals, non-treated and all BCG- or *M. brumae*-treated mice, in multidimensional space. **(B)** The same opt-SNE maps with the expression intensity using panel 1 antibodies and **(C)** using panel 2 antibodies in bladders from mice treated with BCG or *M. brumae* grown in different culture media **(D)** Overlay of 20 gated cell populations on to opt-SNE plots for both antibody panels. **(E)** Heatmaps showing expression intensity in all the populations gated in D) for all the markers using both antibody panels. Color-coded with dark green for higher expression and light green for lower expression. **(F)** Heatmaps displaying frequency for all the populations gated in D) among bladders from all animal groups using panel 1 and **(G)** using panel 2 antibodies. **(H)** Volcano plots showing adjusted p values versus log (Fold Change; FC) for immune populations in different groups of animals using panel 1 and **(I)** panel 2 antibodies. Populations whose expression is significantly different between group of animals with a adjusted p < 0.05 or p < 0.00 are shown in green. The red line denotes a p value of 0.001 or 0.05.

To further explore the global immune profile of the bladder tumor microenvironment after intravesical mycobacterial treatment, computational flow cytometric analysis was performed. We generated a two-dimensional opt-SNE plot to dimensionally reduce the complete multiparametric dataset. Unlike conventional biaxial plots and sequential analysis of pairs of markers, the opt-SNE map captured and summarized the interrelationship between all the markers in an unbiased, data-driven manner. Using two antibody panels (described in **Supplementary Table 1**) and this method, we observed marked differences in the spatial cell distribution, visualized as different patterns of colours, among all groups of animals (**Figure 2A**) and in animals treated with BCG or *M. brumae* grown in different culture media (**Figures 2B, C**, antibody panels 1 and 2, respectively). A more marked effect was observed when infiltrating non-T-cells were analysed (**Figure 2C**). To provide more specificity to our analysis, we subdivided the opt-SNE plot of each panel into 20 spatially distinct subpopulations (**Figure 2D**). Heatmaps revealed the differential expression of all markers in the 20 subpopulations (**Figure 2E**). In panel 1, ten populations were CD3^+^ T-cells, among which six were CD4^+^ T-cells. Population 18 (P18) corresponded to naïve cells (CD62L^+^CD44^-^), P13 represented natural killer T (NKT) γδ cells (NK1^+^γδ TCR^+^), and the other populations, which were all CD44^+^, could be considered different subsets of memory CD4^+^ T-cells. Within the identified CD8^+^ T-cells, memory and NKT CD8^+^ T-cells (P12 and P17, respectively) contributed to the tumor microenvironment. The CD3^-^ compartment was composed of several populations with variable expression of different markers, including natural killer (NK) cells (NK1^+^, P20) and innate lymphoid cells (ILCs, expressing CD127 and high levels of CD25; P1). In panel 2, nine CD3^+^ populations were identified, three of which had high expression of Ly6C (P3, P15 and P19). Within lymphoid non-T-cells (CD3^-^), two B-cell populations (B220^+^) were identified (P16 and P1). Nine populations were assigned as myeloid cells (CD11b^+^), including dendritic cells (DCs, CD11c^+^; P9, P12 and P18), polymorphonuclear cells/granulocytic myeloid-derived suppressor cells (PMNs/gMDSCs, Ly6C^+^Ly6G^+^; P5), macrophages (F4/80^hi^; P10) and different monocyte populations (P2, P4, P5 and P11). These data highlight the high heterogeneity in the bladder tumor microenvironment after mycobacterial treatment. In concordance with the different spatial cell distributions observed in BCG- or *M. brumae*-treated mice (**Figure 2A**), clear differences were observed when the frequencies of the different populations were plotted as a heatmap (**Figures 2F, G**, upper panels; corresponding to antibody panels 1 and 2, respectively). Seven and four populations identified with panels 1 and 2, respectively, were significantly enriched in BCG-treated mice compared with *M. brumae*-treated mice, with most of them being CD3^+^ populations (**Figures 2H, I**, left part of volcano plots). In contrast, seven and five populations were significantly enriched in *M. brumae*-treated mice (in panels 1 and 2, respectively), with most of them being CD3^-^ populations (**Figures 2H, I**, right part of volcano plots). The heatmaps also showed different global immune profiles for animals treated with BCG or *M. brumae* grown in different culture media showing variations in the cell envelope lipid composition. *M. brumae*-A60 produced a more differential composition of infiltrated populations (**Figure 2F, G**, lower panels, panels 1 and 2, respectively), with a lower proportion of naïve CD4^+^ T-cells (P18; **Figure 2H**, right panel, and **Supplementary Figure 5**, top panels) and significant enrichments or reductions in other populations (**Figure 2I**, left panel, **Supplementary Figure 5**, bottom panels). Of note, two populations that were consistently and significantly reduced with *M. brumae*-A60 treatment compared to the other *M. brumae* treatments were P5 (PMNs/gMDSCs, Ly6C^+^Ly6G^+^) and P4 (monocytes, Ly6C^+^ cells) (**Figure 2I**, **Supplementary Figure 5**, bottom panels). These data suggest that modulation of the global immune infiltration, rather than the quantity of infiltrated cells, could play a critical role in improving intravesical treatment efficacy and survival.

### BCG and *M. brumae* treatments induce differential lymphoid T and non-T-cell bladder immune cell infiltration profiles, whereas mycobacterial culture conditions have a major impact on the tumor B-cell composition

**Figure 3:**
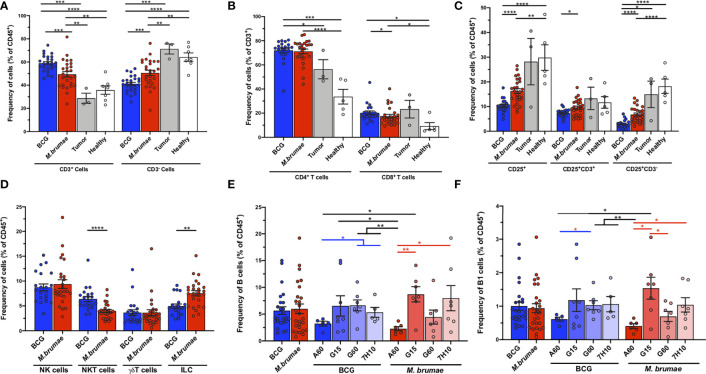
T- and non-T infiltrating cells into the bladder in different groups of mice. Frequencies of bladder infiltrating **(A)** CD3^+^ and CD3^-^ cells; **(B)** CD4^+^ and CD8^+^ T-cells; **(C)** total CD25-expresing cells, CD3^+^CD25^+^ and CD3^-^CD25^+^ cells; **(D)** NK, NKT, TCRγδ^+^ T-cells and ILC; **(E)** B-cells; and **(F)** B1 cells in different groups of mice. Frequencies are given in healthy mice (white bars and dots), untreated tumor-bearing mice (grey bars and dots) and mycobacteria treated-tumor bearing mice, either using BCG (blue bars and dots) or *M. brumae* (red bars and dots) as percentages of the total live CD45^+^ infiltrating immune cells. Data represent the mean (bars) and ± SEM (error bars) and each dot represent an individual mouse. Statistically significant differences between the different BCG and *M. brumae* mycobacteria (intrastrain) are shown as blue and red lines, respectively. Interstrain differences or with the untreated groups (healthy and tumor) are shown in black. Differences were tested using Mann-Whitney U nonparametric test. *p ≤ 0.05; **p ≤ 0.01; ***p ≤ 0.001; ****p ≤ 0.0001.

To further elucidate the heterogeneity of the immune microenvironment in bladder tumors after different treatments, traditional biaxial gating strategies based on 1-3 surface markers were used. Compared to healthy and untreated tumor-bearing mice, mice treated with instillation of mycobacteria showed strong infiltration of CD3^+^ cells into the bladder. In parallel with the absolute infiltration results, BCG treatment recruited larger percentages than *M. brumae* treatment that, on the contrary, had an enriched CD3^-^ cell infiltration (**Figure 3A**). Bladders from mycobacterium-treated mice showed increased percentages of both CD4^+^ T-cells and CD8^+^ T-cells compared to bladders from healthy mice (**Figure 3B**). The CD4^+^ T-cell proportions were similar between BCG- and *M. brumae*-treated mice, although the BCG-treated tumors were significantly enriched in CD8^+^ T-cells compared to the *M. brumae*-treated tumors (**Figure 3B**). These data corroborated the results obtained with the computational unsupervised opt-SNE analysis (**Figures 2F, H**, P17 population). Considering the expression of the activation marker CD25, bladder tumors from *M. brumae*-treated mice were enriched in CD25^+^ populations, both CD3^+^ cells and CD3^-^ cells, compared to those from BCG-treated animals (**Figure 3C**). No significant differences were observed between the groups treated with each mycobacterium grown in different culture media (data not shown). The infiltration of NK and TCRγδ^+^ T-cells remained constant regardless of the mycobacterial treatment administered (**Figure 3D**). However, NKT cells were enriched in BCG-treated mice, whereas tumors from *M. brumae*-treated mice contained more infiltrating ILCs (**Figure 3D**). No significant differences in infiltrated immune cells were observed among each mycobacterium grown in different culture media (data not shown). The B-cell lineage contribution was also evaluated. The total B-cell (CD3^-^B220^+^) and peritoneal B1 subset (CD3^-^B220^+^CD11b^+^) frequencies did not show any significant difference when comparing BCG- or *M. brumae*-treated mice (**Figures 3E, F**, gating strategy **Supplementary Figure 2**). Nevertheless, the B-cell response in the tumor microenvironment was strongly influenced by the antigenic changes induced by the culture conditions in which the mycobacteria were grown. The B-cell and B1 populations were found to be significantly reduced in animals treated with both mycobacteria grown in A60 medium (**Figures 3E, F**).

### BCG and *M. brumae* as well as their cell envelope composition alter the maturation profile of tumor-infiltrating CD4^+^ and CD8^+^ T-cells

**Figure 4:**
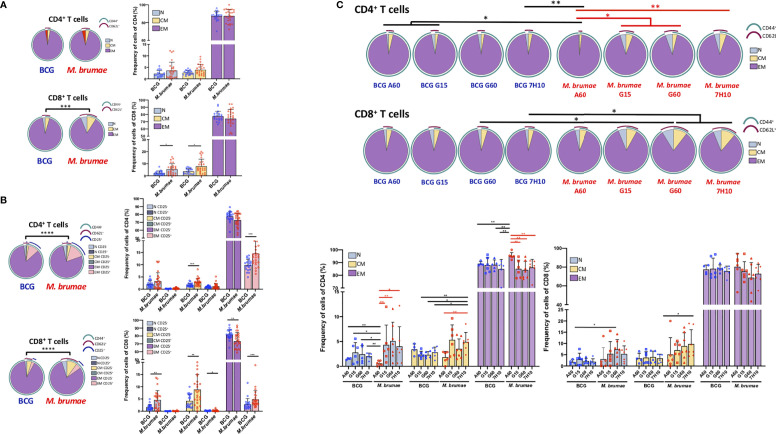
CD4^+^ and CD8^+^ T -cell maturation profiles of bladder infiltrating cells differ after different mycobacteria treatments. Immune maturation profile in BCG- and *M. brumae*-treated mice in **(A)** CD4^+^ and CD8^+^ T-cells and in **(B)** CD4^+^ and CD8^+^ T-cells expressing CD25. The differential marker expression was analyzed by boolean gating. **(C)** CD4^+^ and CD8^+^ T-cell maturation profile in bladder tumors from mice treated with BCG and *M. brumae* cultured in different media. Pie charts illustrate relative proportions of each of the different subsets in the different group of animals. Arcs show population makeup and overlap within pie slice. Statistical analyses of the global maturation profiles were performed by partial permutation tests using the SPICE software (*p < 0.05, **p < 0.01, ***p < 0.001****p < 0.0001). Bar graphs indicate percentages of the different populations within CD4^+^ or CD8^+^ T-cells. Data represent the mean (bars) and ± SEM (error bars) and each dot represent an individual mouse treated with BCG (blue dots) or *M. brumae* (red dots). Statistically significant differences between the different *M. brumae* mycobacteria are shown as red lines. Interstrain differences are shown in black. Differences were tested using Mann-Whitney U nonparametric test. *p ≤ 0.05; **p ≤ 0.01; ***p ≤ 0.001. N: naïve cells (CD62L^+^CD44^-^); CM: central memory cells (CD62L^+^CD44^+^) and EM: effector memory cells (CD62L^-^CD44^+^).

The maturation profile of tumor-infiltrating lymphocytes after treatment was evaluated (gating strategy **Supplementary Figure 1**). Cells were phenotypically identified as naïve (T_N_, CD62L^+^CD44^-^), central memory (T_CM_, CD62L^+^CD44^+^) and effector memory T-cells (T_EM_, CD62L^-^CD44^+^). Infiltrating CD4^+^ and CD8^+^ lymphocytes were primarily composed of T_EM_ cells in all treated animals (**Figure 4A**). No significant differences were observed in the CD4^+^ T-cell maturation distribution of infiltrating cells when all the animals treated with BCG were compared with those treated with *M. brumae* (**Figure 4A**, upper panel). However, the maturation profile of infiltrating CD8^+^ T-cells in the bladder was significantly different between animals treated with each mycobacteria species, with larger frequencies of T_N_ and T_CM_ cells observed after *M. brumae* treatment compared to BCG treatment (**Figure 4A**, lower panel). When the expression of CD25 was included in the analysis by using a Boolean gating strategy, striking differences in the maturation profiles of both CD4^+^ and CD8^+^ T-cell populations were found (**Figure 4B**). An enrichment in CD25^+^ T_EM_ cells with an accumulation of CD25^-^ T_N_ and T_CM_ cells was observed in *M. brumae-*treated mice compared with BCG-treated mice (**Figure 4B**). Regarding the effect of culture conditions, *M. brumae*-A60-treated animals showed a different maturation profile for infiltrating CD4^+^ T-cells than the rest of the animals, where the naïve/memory phenotype distribution of CD4^+^ T-cells was sharply skewed towards an effector phenotype (**Figure 4C**, upper pie charts and left bar graph). A similar tendency was observed for the CD8^+^ T-cell profile, although no significant differences were reached (**Figure 4C**, lower pie charts and right bar graph).

### Immunosuppressive tumor microenvironment is modulated by changes in mycobacterial culture conditions

**Figure 5:**
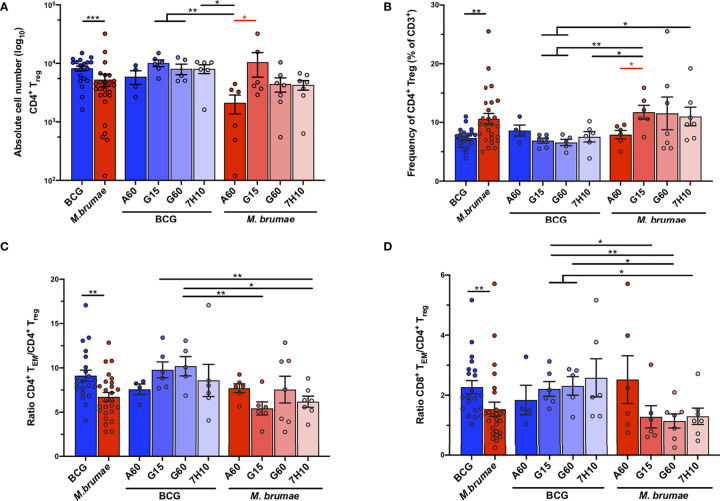
Changes in the infiltration of CD4^+^ T_regs_ after BCG and *M. brumae* treatment. Absolute counts **(A)** and percentages **(B)** of infiltrating CD4^+^ T_regs_ **(**CD3^+^CD4^+^CD25^+^CD127^-^). **(C)** ratio of CD4^+^ T_EM_ and **(D)** CD8^+^ T_EM_ to CD4^+^ T_reg_ cells. Data are shown in BCG- and *M. brumae*-treated tumor-bearing mice pooled or divided by groups according to the medium used to growth mycobacteria. BCG (blue bars and dots). *M. brumae* (red bars and dots). CD4^+^ T_EM_: CD3^+^CD4^+^CD44^+^CD62L^-^. CD8^+^ T_EM_: CD3^+^CD8^+^CD44^+^CD62L^-^. Statistically significant differences between the different *M. brumae* mycobacteria are shown as red lines. Interstrain differences are shown in black.Data represent the mean (bars) and ± SEM (error bars). Each dot represent an individual mouse. Differences were tested using Mann-Whitney U nonparametric test. *p ≤ 0.05; **p ≤ 0.01; ***p ≤ 0.001.

Regulatory T-cells (T_reg_) play crucial roles in the regulation of antitumor immunity (23) The absolute cell number of CD4^+^ T_reg_ (defined as CD3^+^CD4^+^CD25^+^CD127^-^, gating strategy **Supplementary Figure 1**) was significantly higher in bladders from BCG-treated mice than in those from *M. brumae*-treated mice (**Figure 5A**), which was in line with the higher absolute infiltration of most cell populations observed after BCG treatment. In this case, a sharp decrease in the total infiltration of these cells was observed in *M. brumae*-A60-treated mice. On the other hand, when T_reg_ frequencies were analysed, a higher relative CD4^+^ T_reg_ frequency was found in *M. brumae*-treated mice than in BCG-treated mice, with the lowest levels observed in *M. brumae*-A60-treated mice (**Figure 5B**). When the balance between effector T-cells and T_reg_ was analysed, both the CD4^+^ T_EM_/CD4^+^ T_reg_ and CD8^+^ T_EM_/CD4^+^ T_reg_ ratios were significantly higher in BCG-treated mice than in *M. brumae-*treated mice (**Figures 5C, D**).

### Mycobacterial treatment shapes the tumor-infiltrating myeloid compartment

**Figure 6:**
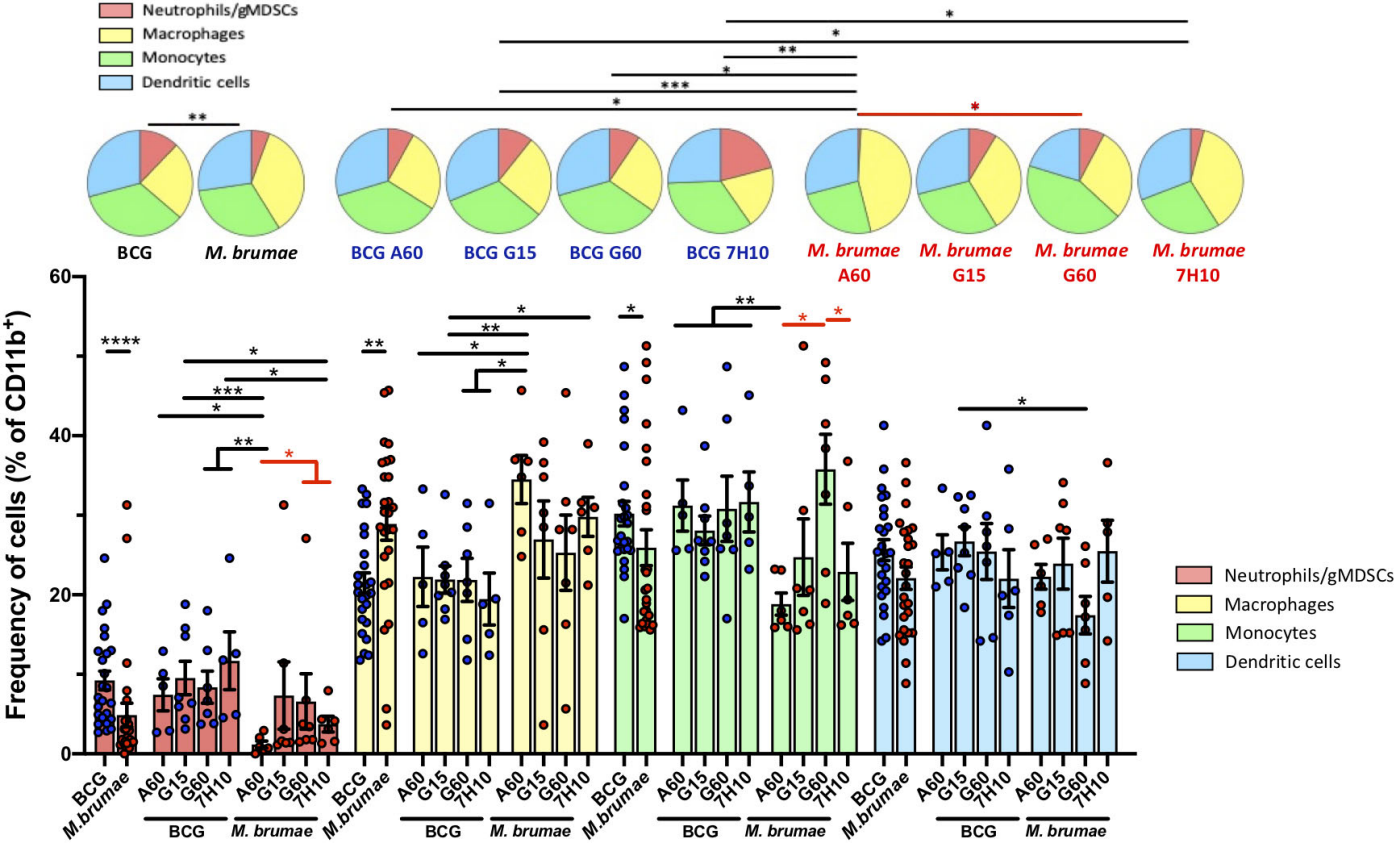
Changes in the tumor myeloid compartment after mycobacteria intravesical treatment. Pie chart representing proportional subpopulations of myeloid cells. CD3^-^CD11b^+^ tumor infiltrating myeloid cells were gated and the relative proportion of neutrophils, macrophages, monocytes and dendritic cells were represented and analyzed using SPICE software. Statistical analyses of the global maturation profiles were performed by partial permutation tests. Bar graphs indicate percentages of the different populations within the total CD11b^+^ cells. Data represent the mean (bars) and ± SEM (error bars) and each dot represent an individual mouse treated with BCG (blue dots) or *M. brumae* (red dots). Statistically significant differences between the different *M. brumae* mycobacteria are shown as red lines. Interstrain differences are shown in black. Differences were tested using Mann-Whitney U nonparametric test. *p ≤ 0.05; **p ≤ 0.01; ***p ≤ 0.001; ****p ≤ 0.0001. Neutrophils (red; CD11b^+^CD11c^-^Ly6G^+^), macrophages (yellow, CD11b^+^CD11c^-^F4/80^hi^), monocytes (green, CD11b^+^CD11c^-^F4/80^lo/int^) and dendritic cells (blue, CD11b^+^CD11c^+^).

We next evaluated the effects of mycobacterial treatments on the composition of myeloid cells in the tumor microenvironment (CD11b^+^ cells) (**Figure 6**, gating strategy **Supplementary Figure 2**). The total distribution of this compartment, composed of neutrophils/gMDSCs, macrophages, monocytes and DCs, was significantly different between BCG- and *M. brumae-*treated mice (**Figure 6**, pie charts). A relative enrichment in macrophages and decreases in neutrophils/gMDSCs and monocytes were observed in tumors from *M. brumae*-treated mice compared with those from BCG-treated mice (**Figure 6**, bar graph). Moreover, the culture conditions for *M. brumae* had a significant impact on the composition of myeloid cells in the tumor microenvironment. *M. brumae*-A60-treated mice showed a skewed myeloid tumor microenvironment in comparison with the rest of the *M. brumae*-treated mice, with the highest decreases in neutrophils and monocytes. DC infiltration was shown to be unaltered by the mycobacterium species or the culture medium used for bacterial growth.

### Monocyte compartment is skewed towards a reparative phenotype after *M. brumae* treatment

**Figure 7:**
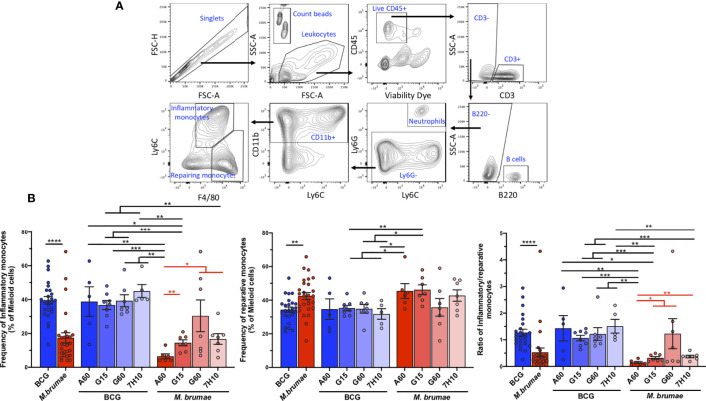
*M. brumae* induce a less inflammatory monocyte phenotype than BCG in bladder tumor tissue and can be modulated by culture conditions. **(A)** FACS gating strategy used to determine inflammatory and reparative monocytes. Doublets were excluded, leucocytes were gated by forward and side scatter and live CD45^+^ tumor infiltrating leukocytes were selected. CD3^-^ cells were gated and the B population (B220^+^ cells) and the neutrophils (Ly6C^+^Ly6G^+^) were excluded. Myeloid CD11b^+^ were selected and the percentage of inflammatory (F4/80^lo/int^Ly6C^+^) and reparative monocytes (F4/80^int/high^Ly6C^-^) were obtained. **(B)** Frequencies of inflammatory monocytes and reparative monocytes within total myeloid cells (CD11b^+^), and inflammatory/reparative ratio from mycobacteria-treated tumor-bearing mice. Data represent the mean (bars) and ± SEM (error bars) and each dot represent a separate mice treated with BCG (blue dots) or *M. brumae* (red dots). Statistically significant differences between the different *M. brumae* mycobacteria are shown as red lines. Interstrain differences are shown in black. Differences were tested using Mann-Whitney U nonparametric test. *p ≤ 0.05; **p ≤ 0.01; ***p ≤ 0.001; ****p ≤ 0.0001.

Mouse monocytes can be subdivided into two major subsets, which are ontologically related but functionally distinct, based on the expression of the antigen Ly6C: classical or inflammatory monocytes (Ly6C^+^) and non-classical resident or reparative monocytes (Ly6C^-^) (24). Therefore, we next evaluated whether the different mycobacterial treatments had an effect on the infiltration of these cells (CD11b^+^Ly6G^-^ F4/80^lo/int^Ly6C^+^ inflammatory monocytes and CD11b^+^Ly6G^-^ F4/80^int/high^Ly6C^-^ reparative monocytes, gating strategy in **Figure 7A**). In tumors from BCG-treated animals, the percentages of inflammatory monocytes were higher than those of reparative monocytes, resulting in inflammatory/reparative ratios >1 (**Figure 7B**). In contrast, a significantly lower proportion of inflammatory monocytes and a higher proportion of reparative monocytes were observed in *M. brumae*-treated animals compared to those treated with BCG, with ratios below 1 (**Figure 7B**). When the effect of culture conditions was taken into account, no significant differences were observed in BCG-treated mice, while *M. brumae* had a major impact on the tumor microenvironment. Remarkably, *M. brumae*-A60-treated mice showed monocyte infiltration mainly limited to the reparative subset, with minimal infiltration of inflammatory monocytes (**Figure 7B**). These data indicate that monocyte phenotype and balance, which involve different functional activities, are strongly dependent on the Mycobacterium species and, in the case of *M. brumae*, are further modulated by mycobacterial cell envelope composition.

## Discussion

In the current study, we showed that in an orthotopic murine model of BC, intravesical treatment with BCG or *M. brumae* altered the bladder tissue microenvironment. The induced immune landscape was species specific and shaped by the mycobacterial cell envelope composition modulated by the culture conditions. In addition, we demonstrated that survival is more dependent upon the balance and quality of the infiltrating immune cells than on the absolute numbers.

Both mycobacterial species were able to induce strong immune infiltration of the bladder, although the immune profiles triggered by the two species differed both quantitatively and qualitatively. The absolute quantification of infiltrating immune cells in the bladder showed that intravesical BCG treatment induced strong immune infiltration, in line with previous reports (10, 19, 20). We observed increases in all the immune cell populations tested, including both T-cells and non-T-cells, innate and adaptive cells, and effector and regulatory cells. This broad immune infiltration triggered by BCG was massive and did not appear to be significantly modulated by the cell envelope composition selected by the culture conditions for the mycobacteria. Conversely, *M. brumae* treatment, which produced more moderate infiltration than BCG, resulted in longer survival. In this case the infiltration was clearly modulated by changes in the cell envelope lipid composition.

The human immune system has developed powerful mechanisms to respond to mycobacterial antigens, among which lipids are known to be potent immune inducers that can modulate both innate and adaptive immune responses through distinct lipid immune receptors (25). Therefore, the lipid profile present in a mycobacterium may have an impact on the global immune response. In our model, a multidimensional immune profiling analysis revealed the high degree of heterogeneity and complexity of the phenotypes of immune cells mobilized into the bladder after intravesical treatment, and notably, the bladder immune landscape induced by the two mycobacterial species or the different antigenic variants differed. These data suggest that the tumor immune microenvironment in BC patients treated with intravesical therapy can be remodelled to improve clinical benefit. The differential global immune infiltration observed in the bladder could be explained by the differential cytokine profiles and levels previously reported to be triggered *in vitro* (11, 22) and *in vivo* (10) by each mycobacteria in the panel; very recently, this infiltration was shown to be influenced by the mycobacterial surface lipid composition (21).

BCG treatment has been associated with an infiltration dominated by CD3^+^ cells, particularly memory (CD44^+^) CD4^+^ and CD8^+^ T-cells (19, 20). In our study, we also observed elevated infiltration of CD3^+^ cells, mainly memory CD4^+^ T-cells, in the bladder of BCG-treated mice. Infiltrating CD4^+^ and CD8^+^ T-cells mostly displayed an effector memory phenotype, and a high proportion of these cells presented an activated phenotype (CD25^+^). These data agree with the immune activation described after *in vitro* exposure of human blood cells to BCG or after immunological evaluation of tissues from patients with NMIBC (17, 26, 27). Notably, compared with BCG treatment, *M. brumae* treatment triggered a more immune-active tumor microenvironment, increasing the proportion of total activated immune cells and producing enrichment of activated CD4^+^ and CD8^+^ T_EM_ cells. Among the mice treated with *M. brumae* variants, *M. brumae*-A60-treated mice displayed a maturation profile distribution more sharply skewed towards an effector phenotype, with a greater impact on the CD4^+^ T-cell population. This increase in the proportion of CD4^+^ T_EM_ cells is relevant, since CD4^+^ T-cells have been described as potent cytotoxic cells (28) and very recently, in bladder tumors, have been linked to the efficacy of immunotherapy (29).

T_reg_ have been associated with a poor prognosis in many cancers, including BC, due to their immunosuppressive functions within the tumor microenvironment (14, 30). In NMIBC, baseline T_reg_ in the tumor prior to BCG treatment have been previously shown to correlate with treatment failure (14). However, in a recent study, Lim et al. reported that although BCG treatment enhanced T_reg_ infiltration, the presence of these cells was not associated with the response to treatment (18). Increasing evidence indicates that improved clinical outcomes and treatment responses are dependent on the intratumoral balance between regulatory and effector cells (29, 31). After mycobacterial treatment, in agreement with previous reports (18, 32), we observed the presence of CD4^+^ T_reg_ in the bladder, with a higher absolute infiltration but lower proportions observed after BGG treatment than after *M. brumae* treatment. Higher CD4^+^ T_EM_ cell/CD4^+^ T_reg_ and CD8^+^ T_EM_ cell/CD4^+^ T_reg_ ratios were also observed in bladders from BCG-treated mice than in those from *M. brumae*-treated mice. These data show that *M. brumae* establishes a more immunosuppressive microenvironment than BCG; however, the antigenic changes in *M. brumae*-A60 are able to equilibrate the balance between effector and regulatory cells in the tumor microenvironment, increasing the percentage of infiltrating T_EM_ cells and decreasing the percentage of T_reg_.

One limitation of our study was that CD4^+^ T_reg_ were identified by the expression of CD25 and the absence of CD127 (32) but no other commonly used markers, such as the described “specific” molecular marker FOXP3, were used (33). The identification of T_reg_ following immune cell activation, as during an immune response to a pathogen, is challenging since conventional T-cells also express CD25 and lose CD127 expression (34). Moreover, accumulating evidence has suggested that FOXP3 expression may not always specifically identify T_reg_, as activated T-cells transiently upregulate FOXP3 without acquiring a T_reg_ phenotype or functions (35). Therefore, in the highly inflammatory tumor microenvironment established after mycobacterial treatment, the quantitative identification of T_reg_ using phenotypic markers could produce an overestimation that does not represent the cells that actually exert a real immunosuppressive function.

In addition to the classical CD3^+^ T-cell response, which forms the core of the adaptive immune system, other lymphoid populations have been described to play important roles in the regulation of antitumor immunity (36). In the context of BC, innate immune recognition of live BCG contributes to immunogenicity, and there are reports indicating the involvement of NK cells, NKT cells, TCRγδ cells and ILCs in antitumor immune responses and associations with a good response to immunotherapy (37). In our model, we found evidence of significant infiltration of the bladder by all these populations, but only the proportions of NKT cells and ILCs were influenced by the mycobacterial species, with a lower proportion of NKT cells and a higher frequency of ILCs in *M. brumae*-treated mice than in BCG-treated mice.

Tumor-infiltrating B-cells are another intratumoral non-T lymphoid population that has been linked to survival in different malignancies, including urothelial BC (38), with both positive and negative outcomes reported depending on the cancer type and the specific B-cell population (39). Here, we showed that total B- and innate-like B1-cells remained unaffected by the Mycobacterium species instilled in the bladder but that interestingly their proportions in immune infiltrates were strongly dependent on the mycobacterial cell envelope lipid composition. This finding agrees with the participation of B-cells in the lipid immune response (40), as demonstrated by the activation of B and B1 cells by lipids from *Mycobacterium tuberculosis* (41). The precise identification and functional relevance of the B-cell subtypes present in our model remain to be determined, as although tumoral B-cell populations have been correlated with a good prognosis in BC (38), high levels of either B-cells or plasma cells infiltration into tumors have also been associated with increased invasiveness (42), significantly worsening patient prognosis.

Different cell types within the myeloid cell lineage have also been shown to be involved in the regulation of immune activity in cancer (43). In agreement with previous reports (17, 28, 44), we found that mycobacterial treatment enhanced the infiltration of myeloid cells compared with no treatment. BCG and *M. brumae* treatments produced markedly different myeloid cell infiltration profiles, which were influenced by the cell envelope composition. *M. brumae* induced a significantly lower proportion of the neutrophil/gMDSC population than BCG, with *M. brumae*-A60 treatment inducing the lowest levels of these infiltrating cells. This population may actually contain both neutrophils and gMDSCs, as these cell types are phenotypically indistinguishable by means of the markers used in our study. Therefore, both cell types must be considered in the analysis and conclusions for tumor infiltration and their immune activities. On the one hand, myeloid-derived suppressor cells (MDSCs) are well known to be recruited, activated and induced by tumor-derived factors and to directly accelerate tumor development, metastasis and neovascularization (45). Furthermore, following migration into the tumor microenvironment, MDSCs attenuate the antitumor reactivity of T-cells and NK cells *via* various mechanisms (46). Recently, one of the antitumoral mechanisms of cisplatin in BC was reported to be dependent on the depletion of gMDSCs. On the other hand, neutrophils found in tumor tissue have been described to be capable of promoting tumor growth and progression, and their presence is often associated with a poor clinical outcome. However, other data point in the opposite direction, indicating that neutrophils can act as effector cells and combat cancer, leading to the eradication of tumor cells (47). BCG instillation results in an early influx of neutrophils that is believed to orchestrate macrophage and T-cell recruitment through the release of chemokines, which is essential for achieving a positive outcome with BCG therapy (48). The low infiltration of neutrophils observed after *M. brumae*, particularly after *M. brumae*-A60, treatment could be one of the causes of the lower total infiltrating immune cells observed in mice treated with these bacteria. Nevertheless, a low number of neutrophils could be beneficial, as antibody-mediated depletion of neutrophils has been reported to result in decreased metastasis in mouse models of liver and breast cancer metastasis and to inhibit cancer progression in a pancreatic ductal adenocarcinoma model (47).

In addition to neutrophils/gMDSCs, tumor-infiltrating macrophages or monocytes can be found in the urine and bladder wall of BCG-treated patients (17, 26, 27), and their presence in bladder tumors prior to BCG therapy has been correlated with an increased recurrence risk (14, 49). In our model, we observed that *M. brumae*, especially *M. brumae*-A60, treatment shaped monocyte infiltration by decreasing classical or inflammatory monocytes with antimicrobial functions (Ly6C^+^) and increasing nonclassical resident or reparative monocytes (Ly6C^-^) with anti-inflammatory and tissue-repairing functions. In *in vivo* wound repair occurring during skeletal muscle repair or after myocardial infarction, there is a biphasic process that first involves the accumulation of inflammatory monocytes and then transitions into reparative monocytes (50). Therefore, after treatment with *M. brumae*, bladder tumor inflammatory monocytes may be converted into anti-inflammatory reparative monocytes that contribute to regulating the balance of inflammatory responses in the tumor microenvironment.

Another limitation of our study is the validation of our results as a predictive power for BC patients receiving mycobacteria treatment. To compare the administration of BCG strains grown in different culture media together with infiltration of different immune populations into bladder tumor, and the treatment outcome will be the basis for next studies. On the other hand, *M. brumae* treatment need to be further evaluated in clinical trials.

Overall, we demonstrate that the global bladder tumor immune microenvironment can be remodelled by changes in both the bacterial species and the mycobacterial cell envelope composition, improving immune infiltration quality rather than quantity, enhancing survival and increasing safety. Our findings indicate the importance of the balance between inflammatory and regulatory/suppressive activity and the ability to effectively manipulate this balance with therapeutic approaches to enhance tumor control.

## Data availability statement

The raw data supporting the conclusions of this article will be made available by the authors, without undue reservation.

## Ethics statement

*In vivo* experiments were performed in accordance with national and European Union legislation regarding the protection of experimental animals, and were reviewed by the Animal Care Committee at the Autonomous University of Barcelona and the Catalonia government (project 9171) and approved by the Departament d'Agricultura, Ramaderia, Pesca, Alimentació i Medi Natural of the Catalan Regional Government. Mice were supervised following a strict monitoring protocol in order to ensure animal welfare, and euthanized, if required. Consent to participate: Not applicable.

## Author contributions

Conceptualization: SG-G, EJ. Performed experiments: SG-G, EG-M. Analyzed the data: JS, VU, CC. Writing–Original Draft Preparation: JS, CC. Funding acquisition: EJ, CC, BC. Review & Editing Preparation: all authors. Visualization Preparation: all authors. All authors have read and agreed to the published version of the manuscript.

## Funding

This work was funded by the Spanish Ministry of Science, Innovation and Universities grant RTI2018-098777-B-I00 (EJ) and FEDER Funds (EJ), Generalitat of Catalunya grant 2017SGR-229 (EJ), Generalitat de Catalunya PhD contracts FI (SG-G). CC is a researcher at the Fundació Institut de Recerca en Ciències de la Salut Germans Trias i Pujol supported by the Health Department of the Catalan Government/Generalitat de Catalunya and ISCIII. This study was partially funded by funds generated at the AIDS Research Institute-IrsiCaixa.

## Acknowledgments

We are grateful to the technical staff from the Estabulari UAB for their outstanding animal management. We thank the IGTP Cytometry Core Facility and Marco Antonio Fernandez for his contribution to this publication. We are grateful to OMIQ for giving us the opportunity to use their platform. **Graphical Abstract** was created with Biorender.com.

## Conflict of interest

The authors declare that the research was conducted in the absence of any commercial or financial relationships that could be construed as a potential conflict of interest.

## Publisher’s note

All claims expressed in this article are solely those of the authors and do not necessarily represent those of their affiliated organizations, or those of the publisher, the editors and the reviewers. Any product that may be evaluated in this article, or claim that may be made by its manufacturer, is not guaranteed or endorsed by the publisher.

## Supplementary material

Supplementary Figure 1 Gating strategy to analyze the phenotype (percentage and absolute number) of leukocytes infiltrating the bladder. Doublets were excluded, lymphocytes were gated by forward and side scatter and live CD45^+^ tumor infiltrating leukoytes were selected for further gating. ILCs were defined as CD3^-^CD127^+^ cells. NK as NK1.1^+^CD3^-^, NKT cells as NK1.1^+^CD3^+^, and T-cells as NK1.1^-^CD3^+^ populations. From the CD3^+^ gate CD4^+^, CD8^+^ and TCRγδ^+^ were defined as CD3^+^CD4^+^, CD3^+^CD8^+^ and CD3^+^TCRγδ^+^ cells, respectively. Regulatory CD4^+^ T-cells were selected from CD4 T-cells as CD25^+^CD127^-^. The different CD4^+^ and CD8^+^ T-cell maturation phenotypes were analyzed using CD62L and CD44 markers as follows: Naïve (N) were defined as CD62L^+^CD44^-^, central memory (CM) as CD62L^+^CD44^+^, and effector memory (EM) as CD62L^-^CD44^+^ cells.

Supplementary Figure 2 Gating strategy to analyze the phenotype (percentage and absolute number) of non-T immune cells infiltrating the bladder. Doublets were excluded, leukocytes were gated by forward and side scatter and live CD45^+^ tumor infiltrating leukocytes were selected for further gating. B-cells were identified as the B220^+^ population gated from CD3^-^ cells. B1 cells (B220^+^CD11b^+^) were selected from the B-cell gate. All non-B-cells (B220^-^) were further gated into CD11b^+^ myeloid cells and, subsequently neutrophils and DCs were analyzed as CD11c^-^Ly6G^+^and CD11c^+^Ly6G^-^, respectively. Finally, CD11c^-^Ly6G^-^ cells were gated into monocytes (F4/80^lo/int^) and macrophages (F4/80^hi^).

Supplementary Figure 3 Survival curves after intravesical mycobacterial treatment. Kaplan-Meier analysis survival of healthy mice, tumor-bearing mice treated with PBS. pooled BCG grown in different culture media, A-60-*M. brumae* and the rest of *M. brumae*. ****p ≤ 0.0001 versus PBS group, log-rank (Mantel-Cox) test.

Supplementary Figure 4 Bladder infiltration of immune cells after different mycobacteria treatments. Absolute counts of different cell populations were quantified by flow cytometry after bladder digestion of mice treated with BCG (blue bars) or *M. brumae* (red bars) grown in different culture media. Data represent the mean (bars) and ± SEM (error bars) and each dot represent an individual mouse. Statistically significant differences between the different BCG and *M. brumae* mycobacteria (intrastrain) are shown as blue and red lines, respectively. Interstrain differences are shown in black. Differences were tested using Mann-Whitney U nonparametric test. *p ≤ 0.05; **p ≤ 0.01; ***p ≤ 0.001.

Supplementary Figure 5 Enriched immune populations into the bladder after *M. brumae*-A60 treatment. Volcano plots showing adjusted p values versus log (FC) for immune populations in different groups of animals using antibodies panel 1 and panel 2. Populations whose expression is significantly different with a adjusted p < 0.05 are shown in green. The red line denotes a p value of 0.05.
