# Supplementary material for: Remodeling the bladder tumor immune microenvironment by mycobacterial species with changes in their cell envelope composition
Source: Front Immunol. 2022 Oct 11;13:993401. doi: 10.3389/fimmu.2022.993401 (PMC9593704; doi:10.3389/fimmu.2022.993401)

Supplementary Figure 1

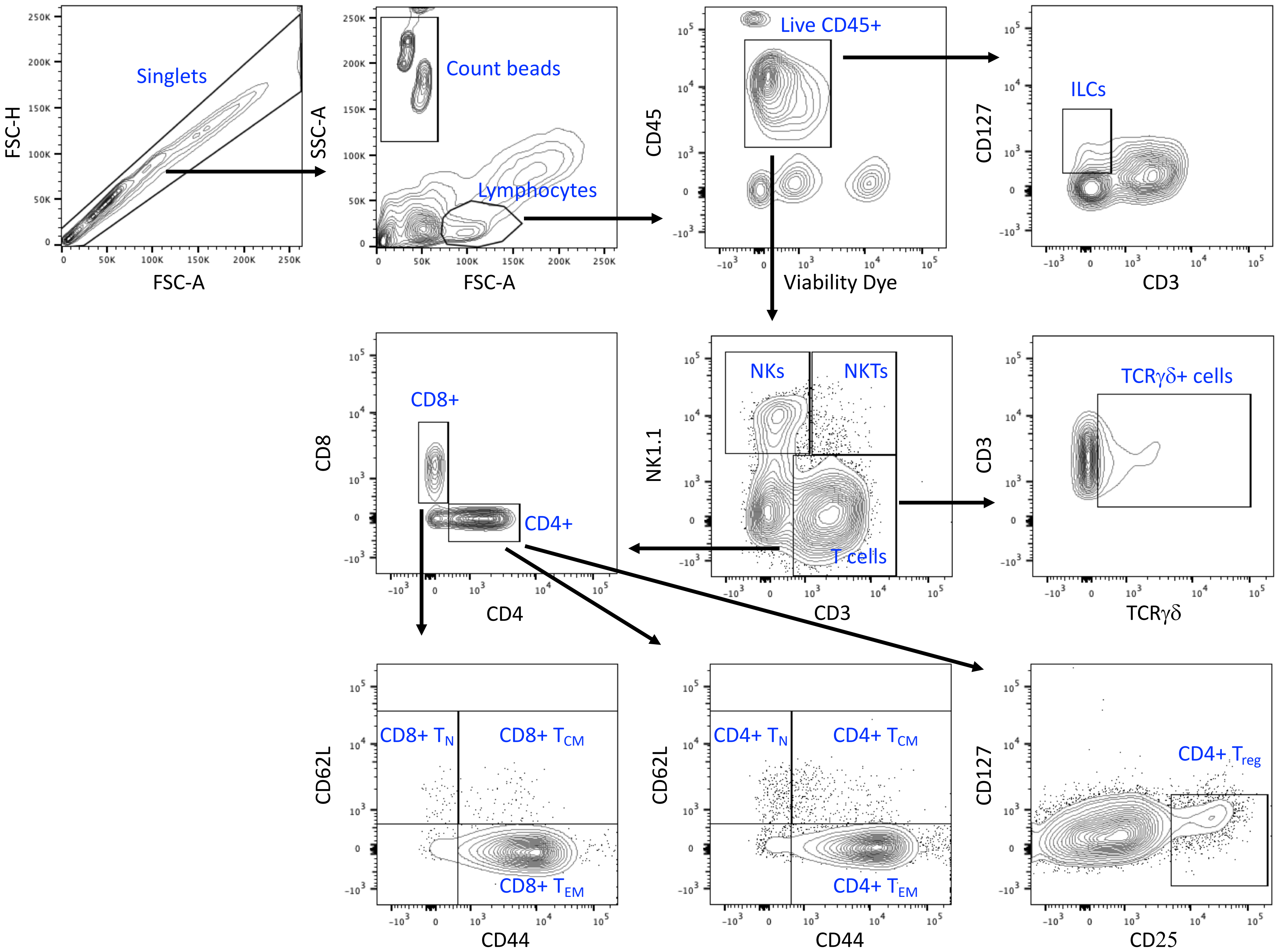

Supplementary Figure 2

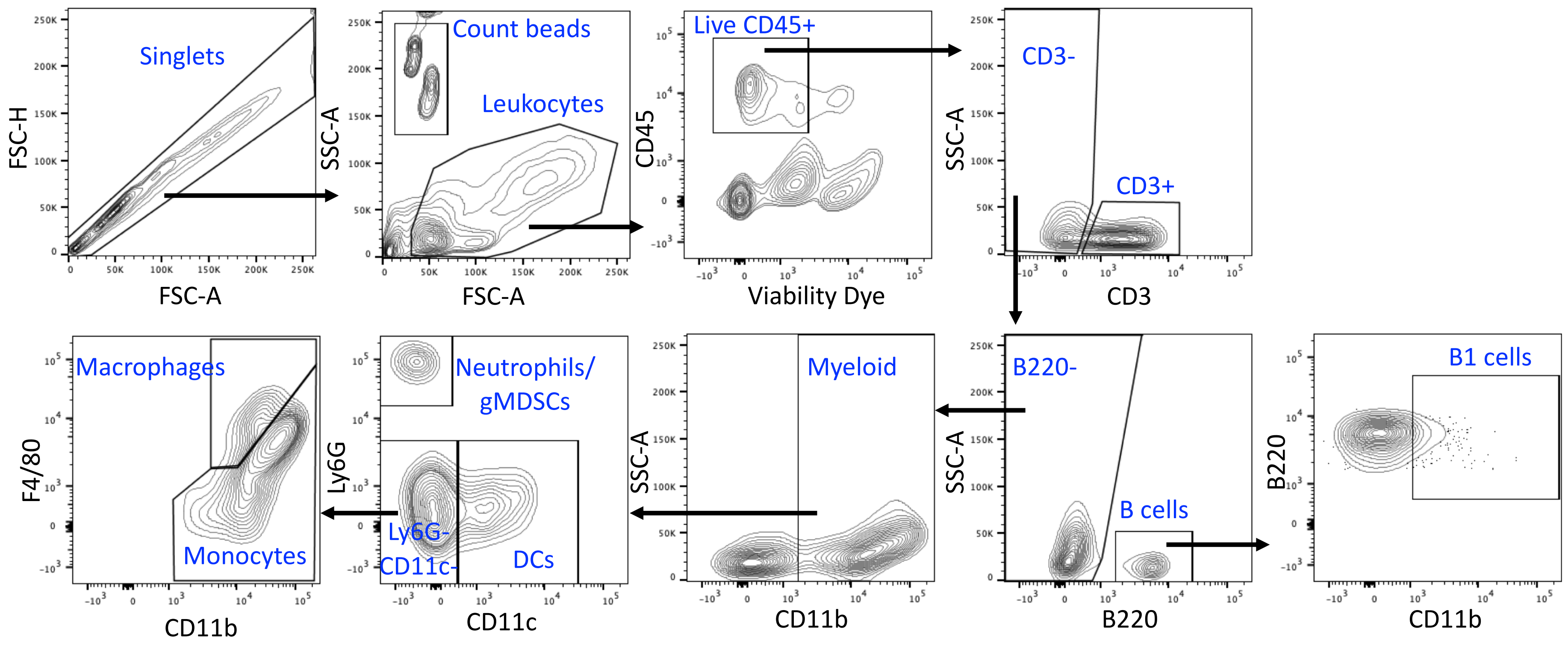

Supplementary Figure 3

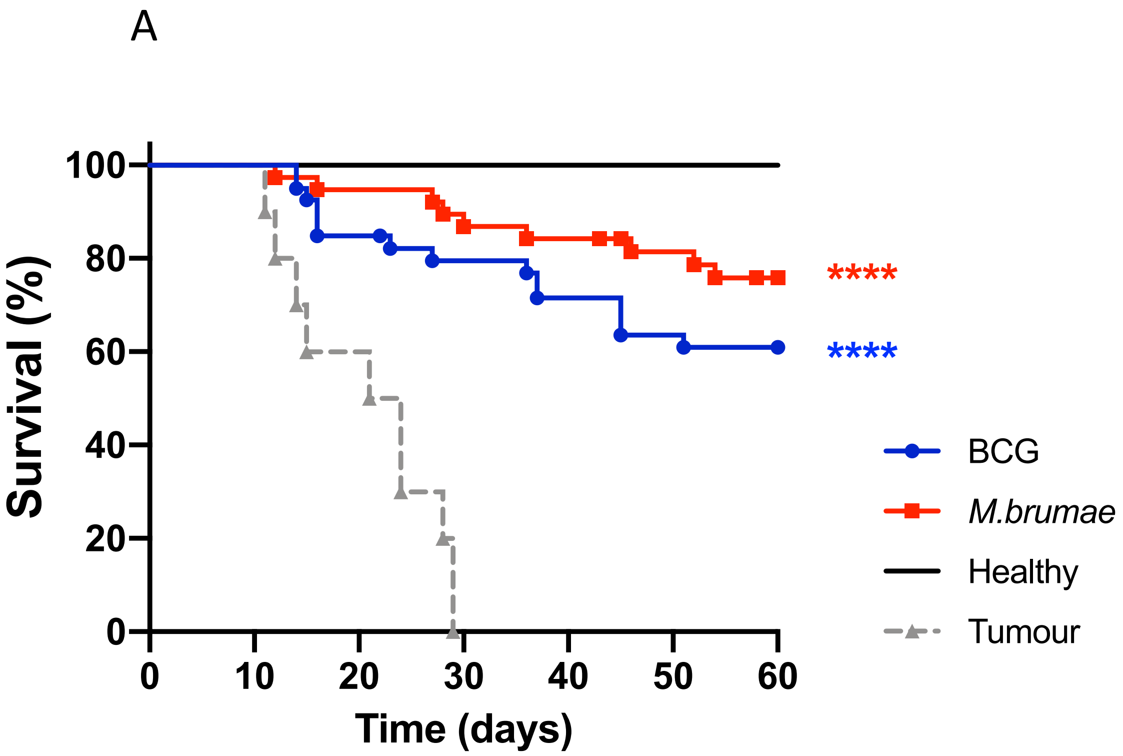

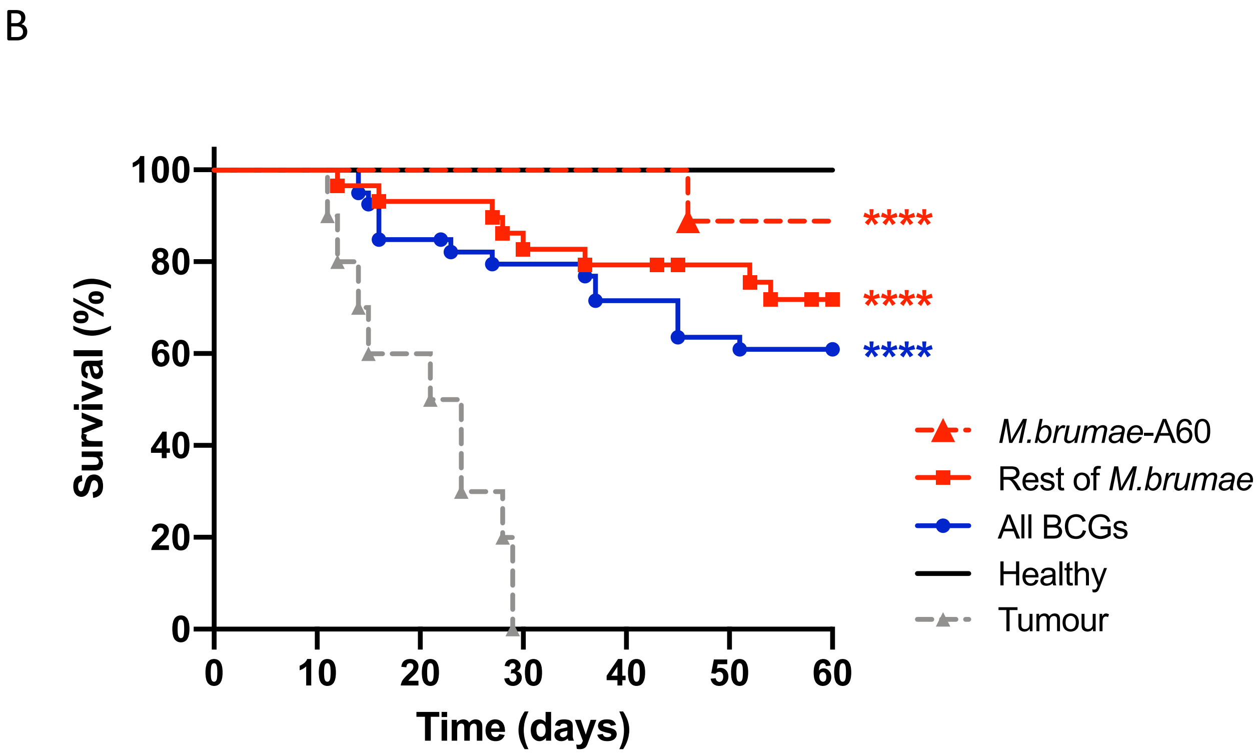

Supplementary Figure 4

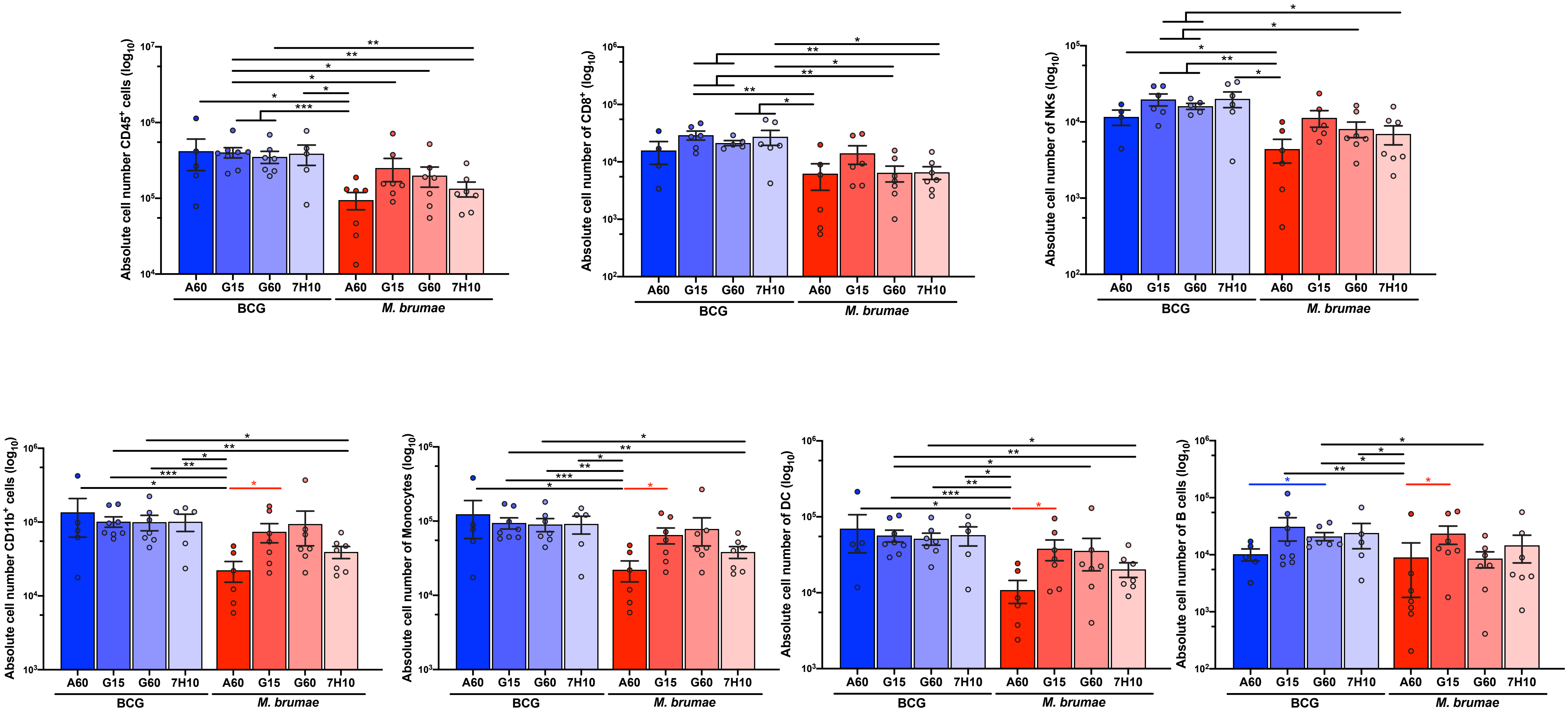

Supplementary Figure 5

Panel 1

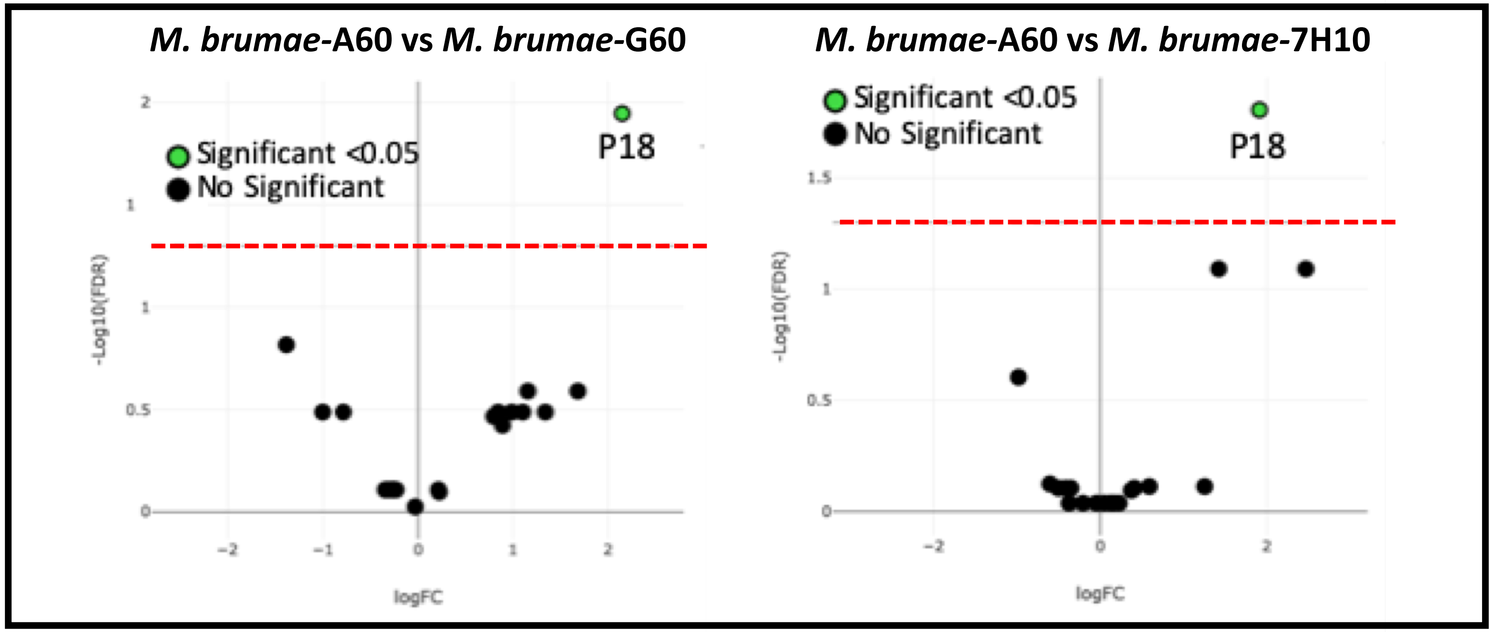

Panel 2

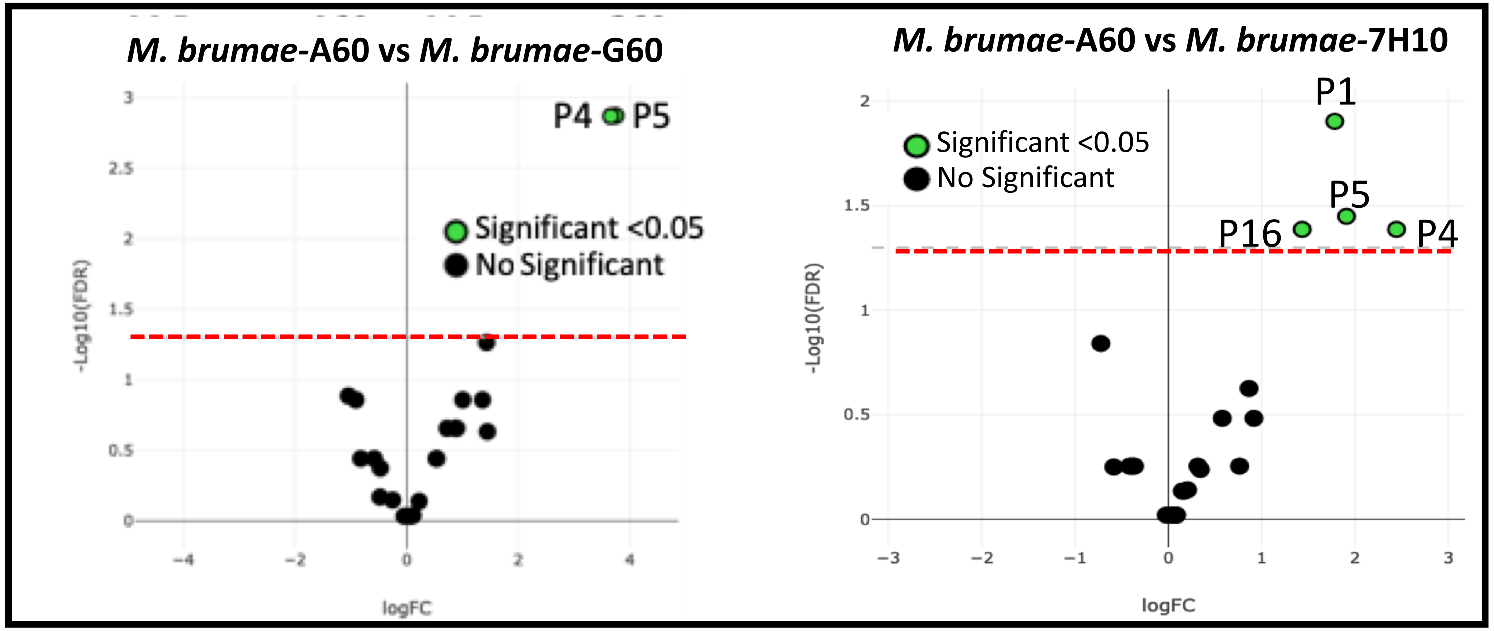

Supplement: Supplementary Figure 1 — Gating strategy to analyze the phenotype (percentage and absolute number) of leukocytes infiltrating the bladder. Doublets were excluded, lymphocytes were gated by forward and side scatter and live CD45+ tumor infiltrating leukoytes were selected for further gating. ILCs were defined as CD3-CD127+ cells. NK as NK1.1+CD3-, NKT cells as NK1.1+CD3+, and T-cells as NK1.1-CD3+ populations. From the CD3+ gate CD4+, CD8+ and TCRγδ+ were defined as CD3+CD4+, CD3+CD8+ and CD3+TCRγδ+ cells, respectively. Regulatory CD4+ T-cells were selected from CD4 T-cells as CD25+CD127-. The different CD4+ and CD8+ T-cell maturation phenotypes were analyzed using CD62L and CD44 markers as follows: Naïve (N) were defined as CD62L+CD44-, central memory (CM) as CD62L+CD44+, and effector memory (EM) as CD62L-CD44+ cells. [file DataSheet_1.pdf]
