# Supplementary material for: Remodeling the bladder tumor immune microenvironment by mycobacterial species with changes in their cell envelope composition
Source: Front Immunol. 2022 Oct 11;13:993401. doi: 10.3389/fimmu.2022.993401 (PMC9593704; doi:10.3389/fimmu.2022.993401)
Supplement: Supplementary file 2 [file DataSheet_2.pdf]

Table supplementary 1. Antibody panels used in the study.

**PANEL 1**			
**Antibody**	**Clone**	**Fluorochrome**	**Source**
CD45	30F11	PerCP	BioLegend
CD3	145-2c11	APC-Cy7	BioLegend
CD4	RM4-5	FITC	BioLegend
CD8	53-6.7	Alexa 700	BioLegend
CD62L	MEL-14	APC	BioLegend
NK1.1	PK136	BV650	BioLegend
CD127	A7R34	BV786	BioLegend
CD44	IM7	PE-Dazzle	BD
CD25	PC61	PE	BioLegend
TCRγδ	GL3	BV421	BioLegend
Aqua Cell Dead			ThermoFisher

**PANEL 2**			
**Antibody**	**Clone**	**Fluorochrome**	**Source**
CD45	30F11	PerCP	BioLegend
CD3	145-2c11	APC-Cy7	BioLegend
CD45R/B220	RA3-6B2	FITC	BioLegend
CD11b	M170	BV650	BioLegend
CD11c	HL3	APC	BD Pharmingen
Ly6G	1A8	BV421	BD Pharmingen
Ly6C	AL-21	PE	BD
F4/80	BM8	BV786	BioLegend
Aqua Cell Dead			ThermoFisher
